# Does Anatomical Contextual Information Improve 3D U-Net-Based Brain Tumor Segmentation?

**DOI:** 10.3390/diagnostics11071159

**Published:** 2021-06-25

**Authors:** Iulian Emil Tampu, Neda Haj-Hosseini, Anders Eklund

**Affiliations:** 1 Department of Biomedical Engineering, Linköping University, 581 83 Linköping, Sweden; neda.haj.hosseini@liu.se (N.H.-H.); anders.eklund@liu.se (A.E.); 2 Center for Medical Image Science and Visualization, Linköping University, 581 83 Linköping, Sweden; 3 Department of Computer and Information Science, Linköping University, 581 83 Linköping, Sweden

**Keywords:** automatic segmentation, artificial intelligence, 3D U-Net, anatomical contextual information, high grade glioma, low grade glioma

## Abstract

Effective, robust, and automatic tools for brain tumor segmentation are needed for the extraction of information useful in treatment planning. Recently, convolutional neural networks have shown remarkable performance in the identification of tumor regions in magnetic resonance (MR) images. Context-aware artificial intelligence is an emerging concept for the development of deep learning applications for computer-aided medical image analysis. A large portion of the current research is devoted to the development of new network architectures to improve segmentation accuracy by using context-aware mechanisms. In this work, it is investigated whether or not the addition of contextual information from the brain anatomy in the form of white matter (WM), gray matter (GM), and cerebrospinal fluid (CSF) masks and probability maps improves U-Net-based brain tumor segmentation. The BraTS2020 dataset was used to train and test two standard 3D U-Net (nnU-Net) models that, in addition to the conventional MR image modalities, used the anatomical contextual information as extra channels in the form of binary masks (CIM) or probability maps (CIP). For comparison, a baseline model (BLM) that only used the conventional MR image modalities was also trained. The impact of adding contextual information was investigated in terms of overall segmentation accuracy, model training time, domain generalization, and compensation for fewer MR modalities available for each subject. Median (mean) Dice scores of 90.2 (81.9), 90.2 (81.9), and 90.0 (82.1) were obtained on the official BraTS2020 validation dataset (125 subjects) for BLM, CIM, and CIP, respectively. Results show that there is no statistically significant difference when comparing Dice scores between the baseline model and the contextual information models (*p* > 0.05), even when comparing performances for high and low grade tumors independently. In a few low grade cases where improvement was seen, the number of false positives was reduced. Moreover, no improvements were found when considering model training time or domain generalization. Only in the case of compensation for fewer MR modalities available for each subject did the addition of anatomical contextual information significantly improve (*p* < 0.05) the segmentation of the whole tumor. In conclusion, there is no overall significant improvement in segmentation performance when using anatomical contextual information in the form of either binary WM, GM, and CSF masks or probability maps as extra channels.

## 1. Introduction

Generally, patients diagnosed with brain tumor undergo radical treatment which can include a combination of surgical tumor resection, radiotherapy, and chemotherapy [1]. In case of surgery, a major factor that influences patient survival and postoperative morbidity is the extent of the resection [1,2]. Treatment planning depends extensively on diagnostic radiology images for the identification of the tumor, key information for balancing the extent of the treatment target with the collateral effects.

MR imaging modalities, such as T1-weighted (T1w), T1-weighted with post-contrast gadolinium enhancement (T1Gd), T2-weighted (T2w), and T2 fluid attenuated inversion recovery (FLAIR), are commonly used for the identification of the tumor [3]. Reliable tools for the extraction of relevant information from the MR images are needed. For this, manual annotation of brain tumors is commonly practiced in clinical routine [4]; however, this is a time consuming and labor-intensive task. Moreover, manual annotation is not objective, with poor agreement between specialists [5]. Automatic methods could overcome these limitations, providing a faster and objective identification of the tumor sub-regions.

Automatic segmentation of brain tumor structures in MR images is challenging and has attracted a great research interest. Among the proposed methods [6], convolutional neural networks (CNNs) have shown state-of-the-art performance, ranking first in the multimodal Brain Tumor Image Segmentation Benchmark (BraTS) challenge during recent years [7]. Given the automatic feature extraction of CNNs [8], the majority of the research is focused on designing network architectures which provide better accuracy for the segmentation task. One of the most popular CNN architectures is U-Net [9], which introduced skip connections between the layers in the network. A plethora of U-Net-like architectures have since then been developed, including, among others, laborious skip connections strategies [10] and attention mechanisms [11]. However, Isensee et al. [12], who obtained top performance using a well-trained U-Net, showed that improving segmentation performance is not only a matter of adjusting the network architecture. The choice of loss function, training strategy, augmentation, and post-processing showed to have a large impact on the segmentation performance.

An emerging topic in artificial intelligence applications, including computer-aided medical interventions, is context-awareness that will allow algorithms to use the information from the surrounding and perform segmentation of images considering the anatomy context [13] and, thus, potentially improve the outcome. The latest literature describes different approaches for context-aware deep learning including auto-context strategies, changing the model architecture, and/or providing additional contextual information during training. Examples of auto-context strategies used to improve model segmentation performance can be seen in References [14,15]. In particular, Reference [15] implemented auto-context in their 3D symmetric fully convolutional neural network by combining multi modal MR images with 3D Haar features with the purpose of improving brain tumor segmentation.

A number of attempts have been made to evaluate the impact of the introduction of context-aware blocks in the model architecture on brain tumor segmentation [16,17,18,19,20]. For example, Pei et al. [19] used a context-aware deep neural network which thanks to a context encoding module between the encoder and the decoder part of the network, helped in overcoming the class imbalance problem that challenges brain tumor segmentation. However, such an implementation does not allow a comparison between the model accuracies with and without the context encoding module; thus, the contribution of context information cannot be assessed.

Another approach for achieving context-awareness is to provide the network with more information [21,22,23,24]. Wachinger et al. [21] included brain spectral coordinates information while training a patch-based deep CNN for brain anatomy segmentation. The authors argued that patches lose context information when propagating through the network, with possible confusion due to the symmetry of the brain. During training, each patch was supplemented with location information obtained from a spectral-based parameterization of the brain volume. Interestingly, the additional information was provided intermediately in the network, concatenating the context information to the feature maps of the initial convolutional layers. In two studies, Shen et al. [22,23] instead added four channels to the input of their fully convolutional network in addition to the four conventional MR modalities. The additional information consisted of symmetry maps computed on all the MR modalities, describing the asymmetry between the brain hemispheres introduced by the tumor. Kao et al. [24] included brain parcellation information during the training of a 3D U-Net as means of location information. In their work, the authors registered the MNI152 1 mm brain parcellation atlas [25] to each BraTS subject, obtaining a mapping of each voxel into one of the defined 69 brain structures.

The aim of this study is to expand this line of thought and investigate if using anatomical contextual information as additional input channels can improve brain tumor segmentation performance considering four aspects: (1) segmentation accuracy when training on multimodal MR images, (2) model training time, (3) compensation for fewer MR modalities available for each subject, and (4) domain generalization. All four aspects are studied also with respect to low grade glioma (LGG) and high grade glioma (HGG) cases independently. Anatomical contextual information is defined in this study as white matter (WM), gray matter (GM), and cerebrospinal fluid (CSF) masks or probability maps obtained automatically using an automatic segmentation tool.

## 2. Materials and Methods

### 2.1. Dataset

The BraTS2020 dataset was used in this work [7,26,27], which contains 369 preoperative multimodal (T1w, T1Gd, T2w, and T2-FLAIR) 3D MR images, from 19 different centers, of both HGG (*n* = 293) and LGG (*n* = 79). Manual annotations of three tumor sub-regions for each case are provided with the dataset identifying the necrotic (NCR) and the non-enhancing tumor core (NET), the enhancing tumor (ET) and the peritumoral edema tissue (ED). The combination of the above annotations, namely the tumor core (TC = NCR ∪ NET ∪ ET), the ET, and the whole tumor (WT = TC ∪ ED), are targets of the segmentation task. A complete description of the BraTS2020 dataset is available in Reference [7]. Only the BraTS2020 dataset was used in this study, since it is a standardized and widely used dataset that allows comparison between methods and literature.

### 2.2. Anatomical Contextual Information

Contextual information in the form of binary WM, GM, and CSF masks and probability maps was obtained using FMRIB’s automated segmentation tool (FAST) [28] applied on the T1w MR volumes, each with normalized and zero-centered intensity. The difference between the FAST masks obtained from the raw T1w and the intensity normalized and zero-centered T1w volumes was minor. Of the total 369 subjects, 92% showed less than 10% difference in voxel classification (WM, GM, or CSF). The intensity normalized and zero-centered volumes were used instead of the raw data, since a preliminary investigation of the proposed method indicated that segmentation quality was lower when using contextual information from raw T1w data compared to when it was obtained from the intensity normalized and zero-centered volumes. As Tudorascu et al. described [29], methods that use spatial priors during the brain anatomy segmentation, such as the methods in the Statistical Parametric Mapping (SPM) [30] or FreeSurfer [31] softwares, may perform poorly on diseased brains that contain deformations. Brain tumors can induce substantial deformation of the brain structures, making the intensity-based FAST tool more suitable for obtaining the contextual information used in this study, even if it is not specificately designed for patients with tumors. An initial qualitative investigation on obtaining the anatomical segmentation through SPM showed that WM, GM, and CSF masks and probability maps lacked detail and were distorted. Given the qualitatively assessed higher quality of the soft tissue masks, and that no spatial priors are used during the anatomical segmentation, FAST was used in this study.

### 2.3. Model

**Figure 1:**
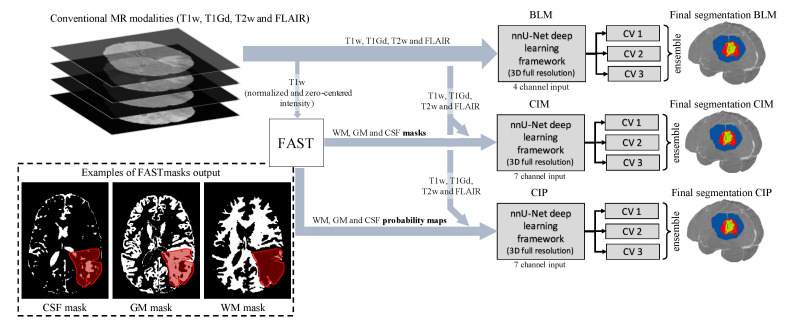
Schematic of the proposed method showing the segmentation for one subject when using all four conventional MR modalities (T1w = T1-weighted, T1Gd = T1-weighted with post-contrast gadolinium enhancement, T2w = T2-weighted, and FLAIR = T2 fluid attenuated inversion recovery) and the 3D U-Net-based deep learning model. By applying FMRIB’s automated segmentation tool (FAST) to the intensity normalized and zero-centered T1w volumes, the contextual information is obtained as white matter (WM), gray matter (GM), and cerebrospinal fluid (CSF) masks. The red patch in the contextual masks shows where the tumor is located. In this region, the contextual masks are distorted. The final segmentation, obtained as an ensemble of three cross-validation (CV) folds and provides regions of tumor core (TC), enhancing tumor (ET), and edema (ED), shown here in green, red, and blue, respectively. BLM is the baseline model, and CIM and CIP are the contextual information models using binary masks and probability maps, respectively.

Figure 1 shows an overview of the methodology, where the nnU-Net deep learning framework [32] was used. There are two reasons for this choice: (1) nnU-Net’s repeated success in the BraTS challenges in the recent years shows the reliability of this framework, which could be difficult to achieve with an in-house model, and (2) this allows reproducibility of the presented investigation. nnU-Net is built upon the 3D U-Net architecture and automatically the tunes network hyperparameters based on the training dataset and hardware available. Among others, the framework tunes the number of convolutional layers, input patch size and batch size [32]. In this study, the 3D full resolution U-Net configuration was adopted, and four NVIDIA Tesla V100 GPUs (32 GB memory) were used for the training. During training, the sum of Dice and cross-entropy loss was minimized using stochastic gradient descent with Nesterov momentum (μ=0.99). The number of training epochs was automatically set to 1000 by nnU-Net, without any early stopping strategies. Each epoch consisted of 250 mini-batches.

### 2.4. Evaluation and Statistical Methods

To investigate if the addition of contextual information has an impact on glioma segmentation performance, three models were trained that differed in the use or not of the contextual information: a baseline model (BLM) with input channels chosen among the four conventional MR modalities provided by BraTS, and two contextual information models both with three additional channels compared to BLM to accommodate the extra information obtained from FAST. One contextual information model used binary WM, GM, and CSF masks (CIM), while the other model used the WM, GM, and CSF probability maps (CIP). A 3-fold cross validation scheme was used to train each setting described below. After training, the segmentation of the test subjects was obtained as an ensemble of the predictions of the three models trained through cross validation. Dice score [26] and 95% Hausdorff distance (HD) [26] on the segmentation targets were obtained through the automatic validation system, on both the official BraTS2020 validation dataset (125 cases) and an independent test dataset (36 cases), described in Section 2.5. The non-parametric Wilcoxon signed-rank test was used to test the null hypothesis of no difference between the baseline model and the contextual information models, at a significance level of 5%. Statistical analysis was performed in IBM^®^ SPSS^®^ (Version 27.0, Armonk, NY, USA, IBM Corp).

### 2.5. Multimodal MR Model Training

To study the effect of anatomical contextual information on segmentation performance, all four conventional MR modalities were used as input to the three models, with CIM and CIP additionally using the anatomical contextual information, as described above. In addition to the official BraTS2020 validation dataset, 36 subjects containing an equal number of HGGs and LGGs were randomly selected from the training dataset as the independent test dataset, with the remaining 333 subjects used for training. The choice of an independent test dataset with control over the tumor grades allows us to investigate the effect that anatomical contextual information has on the segmentation of LGGs and HGGs independently. Moreover, to understand the impact of contextual information on the model training time, the validation loss curves saved by nnU-Net were analyzed a posteriori for these models. Training was considered finished when the validation loss did not improve over 50 epochs.

### 2.6. Compensation for Fewer MR Modalities

To explore if anatomical contextual information could compensate for the missing information when only one MR modality is used as input, the three models were trained and tested, similarly as in Section 2.5, with only T1Gd provided instead of four MR images per subject. T1Gd was selected, among the other MR modalities, given that (1) it provides contrast of the tumor core region compared to the surrounding healthy tissue [7] and (2) because T1w is already used by FAST to obtain the anatomical contextual information.

### 2.7. Domain Generalization

Finally, to investigate if the addition of contextual information improves domain generalization, the three models were trained on BraTS cases from a single institute and tested on data from a variety of institutes. In particular, a total of 69 (35 LGGs and 34 HGGs), among the 369 cases, were identified to originate from one of the 19 institutes that contributed to the BraTS2020 dataset. Identification of the institutes was possible using the information from References [7,27] and the BraTS name mapping information. Models were trained using all conventional MR modalities, and the 69 cases were excluded from the independent test dataset.

## 3. Results

**Figure 2:**
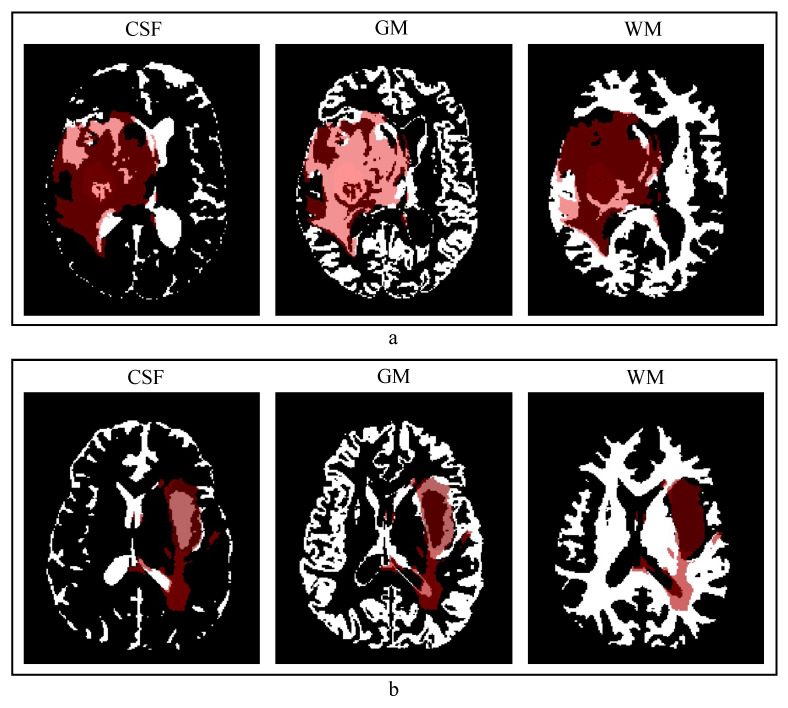
Examples of contextual information obtained using FMRIB’s automated segmentation tool are shown for two cases in an axial slice with the tumor annotation overlay in red. From left to right, cerebrospinal fluid (CSF), gray matter (GM), and white matter (WM) masks. (**a**) A case where the soft tissue masks are highly distorted; (**b**) a case where masks are only distorted in the tumor region.

An example of contextual information obtained using FAST can be seen in Figure 2, where the cross-section of CSF, GM, and WM masks are shown for two subjects. By visually inspecting the FAST results, the soft tissue segmentations are descriptive of the brain WM, GM, and CSF structures, with the masks and probability maps being distorted only in the regions where the tumor is located or proximal to it.

### 3.1. Segmentation Accuracy for Multimodal MR Model Training

**Figure 3:**
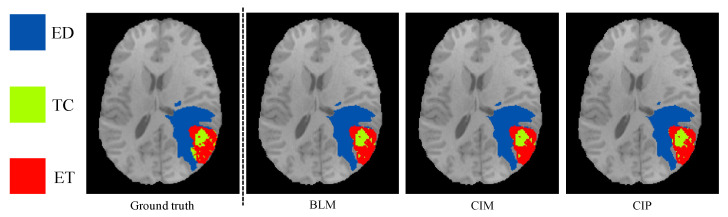
Segmentation results obtained for one of the independent test subjects and for the three models (BLM = baseline model, CIM = contextual information model using binary masks, and CIP using probability maps) are shown as colored labels overlaid on a T1-weighted axial slice. Tumor core (TC), enhancing tumor (ET), and edema (ED) are shown in green, red, and blue, respectively. Dice scores for BLM, CIM, and CIP for the presented test case are 92.54, 92.53, and 92.33, respectively.

**Figure 4:**
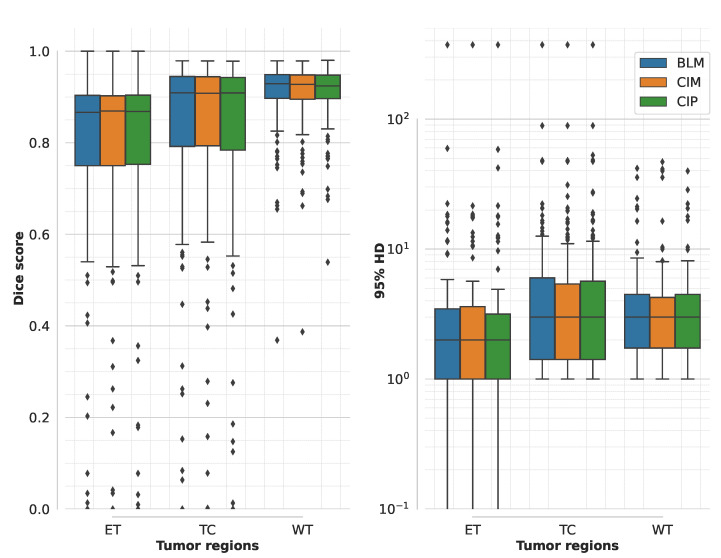
Boxplot of Dice scores and 95% Hausdorff distance (HD) of different models trained on multimodal MR images computed for the official BraTS2020 validation dataset (125 subjects). Boxplots show median and range (with box showing 25% and 75% quantiles) for each model (BLM = baseline model, CIM = contextual information model using binary masks, and CIP using probability maps) and segmentation target (ET = enhancing tumor, TC = tumor core, and WT = whole tumor). The statistical analysis showed no significant difference (*p* > 0.05) when comparing CIM and CIP with BLM.

An example of segmentation results in an axial slice for one of the independent test samples for BLM, CIM, and CIP models when using all the available MR modalities is shown in Figure 3. Performance of the three models on the official validation dataset (125 subjects) is shown as boxplots in Figure 4, where Dice scores and 95% HD are reported for all segmentation targets. Dice scores’ median values (mean) across target regions were 90.15 (81.85), 90.17 (81.87), and 90.04 (82.06), for BLM, CIM, and CIP, respectively. Table 1 summarizes the median Dice scores and and 95% HD obtained on the independent test dataset for LGG and HGG cases separately, showing that HGGs are overall better segmented than LGGs. When comparing CIM and CIP to BLM across the different tumor regions, no statistically significant difference (*p* > 0.05) in Dice scores was found when analyzing the results from both the independent test dataset and the official BraTS2020 validation set. Moreover, no significant difference (*p* > 0.05) was observed when comparing the effect of contextual information in segmenting LGG and HGG cases separately, with HGG showing slightly lower *p*-values. When looking at the cases that showed at least 5% improvement in mean Dice score when using anatomical contextual information, it could be seen that the enhancing tumor region was better segmented. Among the subjects in the independent test dataset, all of those with improved mean Dice score (5.6% of the total subjects) were LGGs, with the contextual information models avoiding false positives for the enhancing tumor region. Table 2 summarizes mean Dice scores across tumor regions for studies that implemented context-awareness by means of auto-context, architectural changes, or additional contextual information. The mean Dice scores obtained in this study are in the same range as results previously reported in literature. Note that the disparity in segmentation performance of the result presented here compared to Reference [32] is due to the fact that, in this work, only the 3D full resolution U-Net configuration was used, instead of a combination of 2D U-Net, 3D low resolution, and 3D full resolution U-Net, that can be trained and ensembled using the nnU-Net framework, at the cost of a longer training time.

### 3.2. Model Training Time for Multimodal MR Model Training

From the a posteriori analysis of the validation loss curves, the baseline model trained 12 and 5 h (46 and 9 epochs) faster than CIM and CIP, respectively, when looking at the average values across the three folds. Average training times and epochs for the three models are summarized in Table 3.

### 3.3. Compensation for Fewer MR Modalities

**Figure 5:**
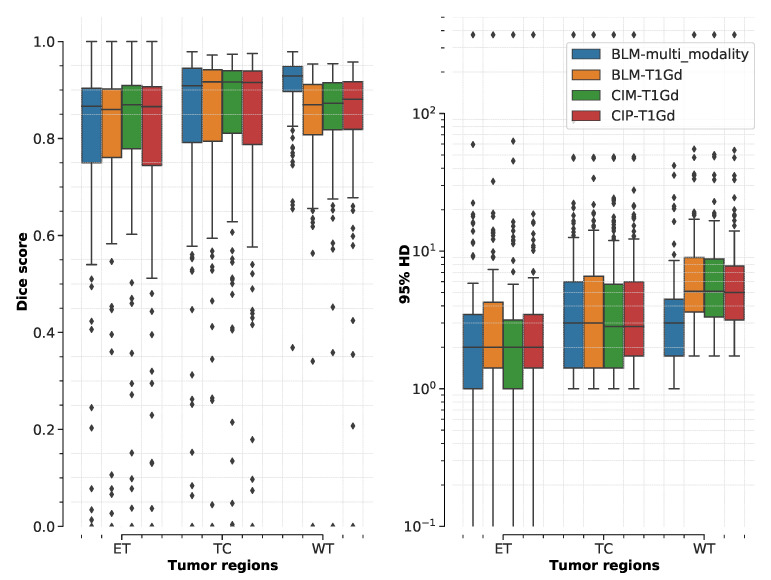
Boxplot of Dice scores and 95% Hausdorff distance (HD) of different models (BLM = baseline model, CIM = contextual information model using binary masks, and CIP using probability maps) trained using only T1-weighted with post-gadolinium enhancement (T1Gd) as single MR modality computed for the official BraTS2020 validation dataset (125 subjects). For comparison, the performance of BLM trained on data from all institutes is presented (blue box). Boxplots show median and range (with box showing 25% and 75% quantiles) for each model and segmentation target (ET = enhancing tumor, TC = tumor core, and WT = whole tumor). By comparing CIM and CIP with the BLM, Dice scores of the WT region show a statistically significant difference (*p* < 0.05).

Segmentation performance results on the BraTS2020 validation set (125 subjects) for BLM, CIM, and CIP when trained using only T1Gd as MR modality are summarized in Figure 5. Dice score values for TC and ET regions are similar when compared to the models trained on all the four MR modalities. The whole tumor region segmentation, on the other hand, shows a decrease in performance that can be attributed to the lack of the contrast in the T1Gd between edema region and surrounding tissue, that is present in FLAIR.

When comparing the models trained only on T1Gd on the BraTS validation dataset, the Dice score for the whole tumor region is significantly improved (*p* < 0.05) for both contextual information models compared to the baseline model (also after Bonferroni correction for multiple comparisons). Considering the results for LGG and HGG cases separately computed on the independent test dataset (36 subjects), no significant difference could be found between the models, not even with respect to the whole tumor region. Median Dice and 95% HD for the independent test set are summarized in Table 4.

### 3.4. Domain Generalization

**Figure 6:**
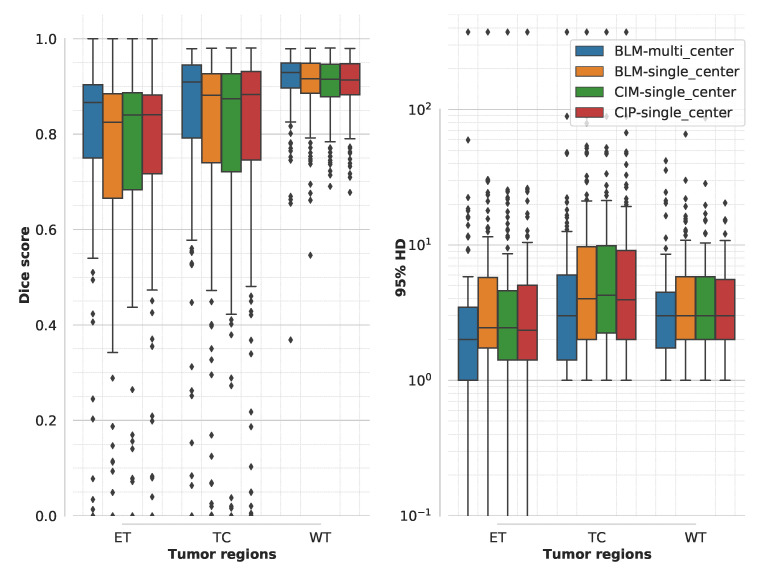
Boxplot of Dice scores and 95% Hausdorff distance (HD) for the different models (BLM = baseline model, CIM = contextual information model using binary masks, and CIP using probability maps) trained on single center data computed for BraTS2020 validation dataset. For comparison, the performance of BLM trained on data from all institutes is presented (blue box). Boxplots show median and range (with box showing 25% and 75% quantiles) for each model and segmentation target (ET = enhancing tumor, TC = tumor core, and WT = whole tumor). The statistical difference was insignificant (*p* > 0.05) when comparing CIM and CIP with BLM.

Dice scores and 95% HD values on the official BraTS validation dataset obtained for the models trained on data from a single institute are summarized in Figure 6. Compared to the baseline model trained on data from all the institutes, performance is lower especially for the TC and ET tumor regions. The drop in performance of all the models trained only on single-center data shows the impact of domain shift between the training and test datasets. The addition of anatomical contextual information does not help in this aspect, since the three models trained on single-center data have similar performances, which are all significantly lower (*p* < 0.05) than the one of the model trained on all the MR data available. Results on the independent test dataset, summarized in Table 5, show a similar trend for both LGG and HGG cases.

## 4. Discussion

The effect of anatomical contextual information on brain tumor segmentation was investigated with respect to segmentation performance, model training, model generalization, and compensation for fewer MR modalities.

### 4.1. Segmentation Accuracy and Training Time for Multimodal MR Model Training

Glioma segmentation performance in this study showed no significant improvement when comparing models trained with the addition of anatomical contextual information as input channels along with the conventional MR modalities. A possible reason for the observed results may be found in how the WM, GM, and CSF information are computed. FAST uses pixel intensity and spatial information for the segmentation. Arguably, this is very similar to what a U-Net architecture is using when trained for semantic segmentation. Thus, it is possible that the network is independently creating a representation of WM, GM, and CSF at some stage during training from the conventional MR modalities, nullifying the additional information. However, providing such information already as input channels did not speed up model training, given that BLM trained faster compared to the contextual information models based on the a posteriori analysis of the validation loss curves. The addition of the contextual information does not improve segmentation performance but instead increases the model convergence time, suggesting that the extra information is not used and makes the segmentation problem harder to solve.

Direct comparison between the obtained results with other works is partially possible, given the differences in testing datasets. The results presented here are in the same range of reported findings of studies that used additional contextual information during model training. Overall, the reported results in literature and the ones obtained in this study show that the inclusion of context-awareness, by means of model architecture changes or additional information as input to the network, has marginal or no improvement on glioma segmentation [15,16,17,18,19,20,22,23,24]. This should not discourage future research on the topic, but instead promote studies that exploit contextual information for brain tumor segmentation by other approaches and perhaps a combination of the currently implemented methods, i.e., context-aware blocks and additional contextual information as input to the network.

### 4.2. Quality of the Anatomical Contextual Information

Another reason for why the model does not use the additional anatomical information may be found in the quality of the WM, GM, and CSF binary masks and probability maps. As shown in Figure 2, the WM, GM, and CSF masks are distorted in the brain region containing the tumor, which may not help the network. One possible way of investigating this aspect is to compare model performance with anatomical contextual information obtained automatically or from manual annotations. However, the amount of time that would be needed for the annotation of WM, GM, and CSF of each subject is exceedingly large making this comparison unfeasible. Another possible approach is to obtain the anatomical information from quantitative MRI (qMRI) [34]. By quantitatively measuring the relaxation times of tissues in the brain, qMRI can provide probability maps for WM, GM, and CSF. In contrast to the automatically generated probability maps used in this study, the ones obtained through qMRI are not derived quantities; thus, the information given in each of the input channels is unique and is not a different version of the same information. This does increase the amount of information that the model can actually use for the segmentation task. Given that the BraTS2020 dataset does not provide qMRI data, this approach remains open for investigation.

### 4.3. Compensation for Fewer MR Modalities

Reducing the MR modalities needed to be acquired could have a positive impact on patient hospitalization experience and on healthcare economy, since a shorter time would be needed for the patient to be in the MR scanner and more patients could be scanned [35]. For this reason, here, it was investigated whether or not the addition of anatomical information could compensate for the decrease in segmentation performance caused by using only one MR modality (T1Gd) as input to the model. Results show that only the segmentation of the whole tumor region is affected by the lack of the excluded MR modalities. This is not surprising since the WT includes the ED region, which is not visible in T1Gd. However, the addition of contextual information marginally improves WT segmentation, suggesting that the WM, GM, and CSF masks help the model to better identify the edema region.

### 4.4. Domain Generalization

Domain shift is a challenge that today’s deep learning models in general have to address when intended for real world applications [36]. In the context of medical image segmentation, models trained on data from a single center or scanner often struggle to retain segmentation performance when tested or used on data that originates from different centers or scanners. As described by Zhou et al. [36], domain generalization is still a challenge that hinders the expansion of deep learning methods for real world applications. Anatomical contextual information has shown no impact on domain generalization, given that the models trained on single center data and with WM, GM, and CSF as extra input channels have suffered from a similar drop in the performance compared to the baseline performance as the model trained without it. A possible reason for this, as discussed above, is that the model is not using the additional anatomical information.

### 4.5. Effect of Contextual Information on LGG and HGG Cases

Overall, anatomical contextual information does not improve segmentation performance when considering high and low grade cases separately. Even if no statistically significant improvement could be found between the baseline and the contextual information models, in some of the LGG cases, the addition of contextual information reduced the number of false positives for the enhancing tumor region. The low number of LGGs in the training dataset, compared to HGGs, could bias the model in always segmenting an enhancing tumor region. However, in LGG cases, this tumor sub-region is missing, which leads to a high number of false positives when the model segments it. When using anatomical contextual information, the model could better discriminate HGG and LGG cases, thus avoiding segmenting the enhancing tumor region that is not present in LGGs. Yet, the statistical comparison does not support this hypothesis, since no statistically significant difference was found between the baseline model and the contextual information models for the cases in this study. Nevertheless, a higher number of LGG test samples are needed to study this effect.

### 4.6. Future Perspectives

A possible approach to investigate is providing contextual information not as additional channels to the input, but after the initial convolutional layers. Wachinger et al. [21] showed improved neuroanatomy segmentation performance when concatenating context information intermediately in the convolutional network. Moreover, the authors also showed that different types of context information affect the performance differently; a combination of spectral and cartesian-based parametrization of the brain volume yielded a better performance than when only one of the two was used, suggesting that they might contain complementary information. Thus, investigation could focus on finding different types of contextual information and their combinations. Future research could also address if using additional MR modalities (e.g., diffusion MR imaging) would improve brain tumor segmentation. The major factor hindering such investigation at the current moment is the lack of a standardized open access dataset which includes extra MR modalities.

## 5. Conclusions

Anatomical contextual information in the form of binary WM, GM, and CSF and probability maps was obtained in this study using the automatic FAST segmentation tool. The addition of anatomical contextual information as extra channels to the network shows no statistically significant difference in tumor segmentation performance when using a standardized 3D U-Net architecture and conventional multimodal MR images as dataset. Only in the case of using one conventional MR modality (T1Gd) did the addition of the anatomical contextual information show to significantly improve whole tumor region segmentation. No statistically significant improvements could be seen when investigating HGG and LGG cases separately, nor when considering model training time, domain generalization, and compensation for fewer MR modalities. Overall, context-aware approaches implemented for brain tumor segmentation in the recent literature show only minor or no improvements. This suggests that effective integration of context awareness in deep learning models for glioma segmentation has yet to be explored.

## Figures and Tables

**Table 1:** Median Dice scores and 95% Hausdorff distance (HD) across target regions (ET = enhancing tumor, TC = tumor core, and WT = whole tumor) for the different models (BLM = baseline model, CIM = contextual information model using binary masks, and CIP using probability maps) trained on multimodal MR images. Results are shown for low grade glioma (LGG) and high grade glioma (HGG) cases independently computed on the independent dataset (36 subjects). There was no significant difference (*p* > 0.05) when comparing CIM and CIP to BLM for neither of LGG and HGG cases. The results in bold correspond to the best performing model in each tissue region.

Grade	Model	Median Dice Score			Median 95% HD		
		[Min, Max]			[Min, Max]		
		TC	ET	WT	TC	ET	WT
LGG (18 cases)	BLM	79.96	36.72	93.28	7.21	39.66	**3.00**
		[0.00, 98.39]	[0.00, 100.00]	[0.00, 97.09]	[1.00, 373.13]	[0.00, 373.13]	[1.00, 373.13]
	CIM	81.95	44.87	**93.51**	**6.98**	**25.18**	**3.00**
		[0.00, 98.38]	[0.00, 100.00]	[0.00, 96.98]	[1.00, 373.13]	[0.00, 373.13]	[1.41, 373.13]
	CIP	**82.27**	**51.27**	93.22	7.64	23.17	3.08
		[0.00, 80.00]	[0.00, 100.00]	[10.08, 97.18]	[1.00, 373.13]	[0.00, 373.13]	[1.00, 18.49]
HGG (18 cases)	BLM	**94.83**	**89.22**	92.79	**1.41**	**1.41**	2.00
		[65.76, 97.56]	[75.54, 96.57]	[86.64, 97.19]	[1.00, 21.47]	[1.00, 3.00]	[1.00, 7.87]
	CIM	94.80	89.16	**92.94**	1.57	**1.41**	**1.87**
		[64.22, 97.37]	[74.74, 96.17]	[86.90, 97.17]	[1.00, 22.03]	[1.00, 3.00]	[1.00, 7.87]
	CIP	94.54	89.12	92.74	1.57	**1.41**	**1.87**
		[67.68, 97.48]	[74.21, 96.55]	[86.96, 97.27]	[1.00, 14.87]	[1.00, 3.00]	[1.00, 7.55]

**Table 2:** Mean Dice scores reported by similar studies using context-aware methods. * identifies the method ranking first in BraTS2020 (not using context-awareness mechanisms). ^1^ obtained via official BraTS test dataset, ^2^ obtained via official BraTS validation datasets, ^3^ obtained from a randomly selected subset of BraTS training data. ET = enhancing tumor, TC = tumor core, and WT = whole tumor, and BLM = baseline model, CIM = contextual information model using binary masks, and CIP using probability maps. The results in bold correspond to the best performing model in each tissue region.

Model	Dataset	Mean Dice Score			Claimed Improvement Using Context Awareness
		TC	ET	WT	
Isensee et al. [33] *	BraTS2020 ^1^	85.95	82.03	88.95	no context-aware mechanism used
Liu et al. [15]	BraTS2017 ^3^	84.00	78.00	89.00	comparison between models with and without context-aware mechanism not available
Liu et al. [18]	BraTS2019 ^2^	85.10	75.90	88.50	
Ahmad et al. [16]	BraTS2020 ^1^	84.67	79.10	89.12	
Chandra et al. [17]	BraTS2018 ^1^	73.33	61.82	82.99	
Pei et al. [19]	BraTS2019/20 ^3^	83.50	**82.10**	89.50	
Shen et al. [23]	BraTS2013 ^3^	71.80	72.50	88.70	2–3% (no *p*-value)
Shen et al. [22]	BraTS2015 ^1^	82.00	75.00	87.00	1.3% (*p*-value < 0.01)
Kao et al. [24]	BraTS2018 ^1^	79.30	74.90	87.50	1–2% (no *p*-value)
Le et al. [20]	BraTS2018 ^2^	**88.88**	81.41	**90.95**	2% (no *p*-value)
BLM	BraTS2020 ^3^ (36 cases)	81.80	67.20	90.80	none
CIM		81.90	77.00	90.10	
CIP		81.80	70.40	90.50	
BLM	BraTS2020 ^2^ (125 cases)	81.60	73.41	90.54	none
CIM		81.61	73.43	90.58	
CIP		81.44	74.05	90.69	

**Table 3:** Average training epochs and time across folds for the different models (BLM = baseline model, CIM = contextual information model using binary masks, and CIP using probability maps).

Model	Average Training Time [hh:mm:ss]	Epochs [250 Mini-Batches Each]
BLM	8:15:11	79
CIM	20:02:39	140
CIP	12:58:57	103

**Table 4:** Median Dice scores and 95% Hausdorff distance (HD) across target regions (ET = enhancing tumor, TC = tumor core, and WT = whole tumor) for the different models (BLM = baseline model, CIM = contextual information model using binary masks, and CIP using probability maps) trained using only T1-weighted with post-gadolinium enhancement as MR modality. Values are shown for low grade glioma (LGG) and high grade glioma (HGG) cases from the independent test dataset. There was no significant difference (*p* > 0.05) for neither of LGG and HGG cases when comparing CIM and CIP with BLM. The results in bold correspond to the best performing model in each tissue region.

Grade	Model	Median Dice Score			Median 95% HD		
		[Min, Max]			[Min, Max]		
		TC	ET	WT	TC	ET	WT
LGG (18 cases)	BLM	**79.99**	**61.08**	**86.65**	9.25	176.67	86.01
		[0.00, 98.06]	[0.00, 100.00]	[0.00, 95,34]	[1.00, 373.13]	[0.00, 373.13]	[0.00, 373.13]
	CIM	78.21	37.93	83.85	**6.42**	37.85	**9.26**
		[0.00, 98.04]	[0.00, 100.00]	[0.00, 95.16]	[1.00, 373.13]	[0.00, 373.13]	[2.00, 373.13]
	CIP	79.58	52.22	83.27	7.87	**23.43**	10.37
		[0.00, 98.21]	[0.00, 100.00]	[0.00, 94.87]	[1.00, 373.13]	[0.00, 373.13]	[2.24, 373.13]
HGG (18 cases)	BLM	**94.41**	89.70	**90.35**	**1.87**	**1.41**	**3.86**
		[87.55, 97.10]	[69.71, 96.29]	[71.90, 94.51]	[1.00, 4.90]	[1.00, 2.83]	[1.73, 15.17]
	CIM	93.90	**89.86**	89.58	**1.87**	**1.41**	3.93
		[66.94, 97.06]	[70.93, 96.41]	[77.27, 94.49]	[1.00, 13.49]	[1.00, 3.00]	[2.00, 13.00]
	CIP	94.10	89.75	89.96	**1.87**	**1.41**	**3.86**
		[63.08, 97.34]	[72.14, 96.45]	[79.54, 94.61]	[1.00, 22.38]	[1.00, 3.00]	[1.73, 10.72]

**Table 5:** Median Dice scores and 95% Hausdorff distance (HD) across target regions (ET = enhancing tumor, TC = tumor core, and WT = whole tumor) for the different models (BLM = baseline model, CIM = contextual information model using binary masks, and CIP using probability maps) trained on single center data. Values are shown for low grade glioma (LGG) and high grade glioma (HGG) cases from the independent test dataset (36 subjects). The statistical difference was insignificant (*p* > 0.05) for either LGG or HGG cases when comparing CIM and CIP with BLM. In bold, the results of the better performing model for each segmentation target.

Grade	Model	Median Dice Score			Median 95% HD		
		[Min, Max]			[Min, Max]		
		TC	ET	WT	TC	ET	WT
LGG (18 cases)	BLM	**78.47**	**60.81**	92.77	**6.40**	**7.64**	2.73
		[0.00, 97.31]	[0.00, 100.00]	[76.48, 97.11]	[0.00, 97.31]	[1.00, 373.13]	[0.00, 373.13]
	CIM	75.99	56.71	93.02	6.71	13.42	2.53
		[0.00, 96.85]	[0.00, 100.00]	[84.68, 97.21]	[1.00, 373.13]	[0.00, 373.13]	[1.41, 12.69]
	CIP	73.53	60.19	**93.29**	6.54	13.98	**2.45**
		[0.00, 97.05]	[0.00, 100.00]	[14.81, 96.94]	[1.00, 373.13]	[0.00, 373.13]	[1.00, 14.78]
HGG (18 cases)	BLM	91.68	85.66	89.30	**2.24**	2.00	**3.23**
		[63.67, 96.15]	[53.57, 94.62]	[76.57, 96,62]	[1.00, 12.37]	[1.00, 6.63]	[1.00, 75.21]
	CIM	90.97	**86.37**	89.13	2.34	2.00	3.67
		[67.18, 96.62]	[53.79, 94.29]	[80.83, 96.84]	[1.14, 10.05]	[1.00, 6.48]	[1.00, 13.64]
	CIP	**92.16**	86.33	**89.41**	2.27	**1.23**	3.38
		[63.55, 96.36]	[54.59, 94.25]	[81.16, 96.63]	[1.41, 13.19]	[1.00, 6.40]	[1.00, 10.20]

## Data Availability

Publicly available datasets were analyzed in this study. This data can be found here: https://www.med.upenn.edu/cbica/brats2020/registration.html.
